# Finding Clocks in Genes: A Bayesian Approach to Estimate Periodicity

**DOI:** 10.1155/2016/3017475

**Published:** 2016-06-02

**Authors:** Yan Ren, Christian I. Hong, Sookkyung Lim, Seongho Song

**Affiliations:** ^1^Department of Environmental Health, University of Cincinnati, Cincinnati, OH 45267-0056, USA; ^2^Department of Molecular and Cellular Physiology, University of Cincinnati, Cincinnati, OH 45267-0576, USA; ^3^Department of Mathematical Sciences, University of Cincinnati, Cincinnati, OH 45221-0025, USA

## Abstract

Identification of rhythmic gene expression from metabolic cycles to circadian rhythms is crucial for understanding the gene regulatory networks and functions of these biological processes. Recently, two algorithms, JTK_CYCLE and ARSER, have been developed to estimate periodicity of rhythmic gene expression. JTK_CYCLE performs well for long or less noisy time series, while ARSER performs well for detecting a single rhythmic category. However, observing gene expression at high temporal resolution is not always feasible, and many scientists are interested in exploring both ultradian and circadian rhythmic categories simultaneously. In this paper, a new algorithm, named autoregressive Bayesian spectral regression (ABSR), is proposed. It estimates the period of time-course experimental data and classifies gene expression profiles into multiple rhythmic categories simultaneously. Through the simulation studies, it is shown that ABSR substantially improves the accuracy of periodicity estimation and clustering of rhythmic categories as compared to JTK_CYCLE and ARSER for the data with low temporal resolution. Moreover, ABSR is insensitive to rhythmic patterns. This new scheme is applied to existing time-course mouse liver data to estimate period of rhythms and classify the genes into ultradian, circadian, and arrhythmic categories. It is observed that 49.2% of the circadian profiles detected by JTK_CYCLE with 1-hour resolution are also detected by ABSR with only 4-hour resolution.

## 1. Introduction

Organisms from cyanobacteria to humans have robust time-keeping mechanisms called biological clocks [1, 2]. In mammals, for example, the suprachiasmatic nucleus (SCN) located in the hypothalamus controls circadian rhythms and coordinates timing information with peripheral clocks. Collectively, these clocks regulate rhythmic physiological behaviors such as body temperature, cardiac repolarization, sleep/wake cycle, and metabolism [3–6]. Autonomous oscillations arise from the interplay of core clock components that form transcriptional-translational feedback loops [7]. As protein levels of clock-transcription factors oscillate, their downstream targets also oscillate. Different clocks (e.g., metabolism and cell cycle) may have different patterns of oscillation and target different output genes. Circadian rhythms cycle with a period of about 24 hours, whereas ultradian rhythms cycle with a period of less than 24 hours, and infradian rhythms cycle with a period greater than 24 hours. It has been shown that circadian clocks regulate both circadian and ultradian rhythms [8].

Circadian rhythms coordinate temporal regulation of other cellular processes. For example, the circadian clock regulates transcriptional activation of Wee1, a critical component in the cell cycle that coordinates timing of cell division [9, 10]. Thus, the study of rhythmic gene expression may reveal individual genes (nodes) or even parts of regulatory networks shared by different cellular processes. Finding and characterizing periodic gene expression are a prerequisite for determining these links amongst different oscillatory processes, such as circadian clock, cell cycle, and metabolic cycles.

A series of gene expression levels observed at a set of different time points is called a gene expression profile, and a rhythmic gene produces a rhythmic profile. In general, it is assumed that a rhythmic gene expression profile is correlated with rhythmic periodicity and hence each gene expression takes the form of a series of cosine curves:

(1) $$\begin{array}{c} Y_{t}=\overset{r}{\underset{k=1}{\sum }}A_{k}\cos\left(\;2\pi \omega_{k}t+\phi_{k}\;\right), \end{array}$$

where *Y*_*t*_ is the observed gene expression at time *t*, *r* is the number of component cosine curves, and *A*_*k*_, *ω*_*k*_, and *ϕ*_*k*_ are the amplitude, frequency, and phase of the *k*th component cosine curve, respectively. Several methods have been developed to estimate periods as well as amplitudes and phases (mathematically) of gene expression profiles. Classical approaches such as Fisher's *G*-test [11] and fast Fourier transform (FFT) [12] perform well in estimating periods for long time series, but those approaches are less effective for short time series. Microarrays are commonly used to investigate changes of gene expressions over a time-course, and 4-hour resolution within a 48-hour time interval is a typical experimental design for circadian studies. In other words, microarray data provide short time series (e.g., with 12 time points) for each gene, which results in likely biased outcomes using either Fisher's *G*-test or FFT algorithms. Another widespread approach, COSOPT [13], effectively provides period estimate only with approximately sinusoidal data [14]. Recently, Hughes et al. [15] introduced the Jonckheere-Terpstra-Kendall (JTK_CYCLE) algorithm that applies the Jonckheere-Terpstra (JT) test to the null distribution of Kendall's tau correlations. JTK_CYCLE is an efficient algorithm to estimate periodicity for long or less noisy time series; however, it is less reliable (as are all other methods) when it is applied to noisy short time series [16]. Yang and Su [17] developed an algorithm of autoregressive spectral estimation regression (ARSER) and showed that ARSER is more effective than Fisher's *G*-test and COSOPT in detecting oscillations in a variety of profile patterns, especially, for the microarray data in short time series. ARSER is useful to detect oscillations of a single category, for example, the circadian rhythms, but it is not efficient to detect multiple periods simultaneously.

In this paper, a new algorithm called the autoregressive Bayesian spectral regression (ABSR) is proposed. Built on ARSER, this ABSR algorithm significantly improves true discovery rate (TDR) and reduces FDR for noisy short time series as compared to JTK_CYCLE and ARSER. One of the features of ABSR comes from the use of posterior probabilities for model selection rather than the Akaike Information Criterion (AIC). In situations where the number of model parameters is large relative to the number of observations (e.g., the number of parameters is about one-half of the number of observations), AIC may fail to select the optimal model [18]. In addition, because AIC depends on the estimates of parameters, model selection by AIC may fail to select the most appropriate model if the parameter estimations are biased [19]. Using posterior probabilities for model selection overcomes the shortcomings of AIC by averaging over the uncertainty in the parameter estimates and leads to a more parsimonious model. Another feature of ABSR is that all possible frequencies in the harmonic models are considered and only the unique dominant frequency is extracted for the period estimate. Hence ABSR is able to classify rhythmic genes by different periods.

In Section 2, we present the model to obtain periodic information from time-course data using ABSR algorithm. In Section 3, simulated data and information theory are used to assess the performance of ABSR, ARSER, and JTK_CYCLE, and these algorithms are applied to existing experimental time-course data from mouse liver. Brief conclusions are discussed in Section 4.

## 2. Methods

### 2.1. Overview

The proposed algorithm, the autoregressive Bayesian spectral regression (ABSR), is developed to identify rhythmic patterns in gene expression profiles. The procedure to obtain periodic information from time-course gene expression data is described below.

Suppose *N* genes are observed in an experiment at time points (1,2,…, *T*) with the same lag, and the observed profiles are considered as time series. Let the observed time series of the *i*th gene be **Y**_*i*_ = (*Y*_*i*1_,…,*Y*_*iT*_)′. The raw profile **Y**_*i*_ is then standardized, denoted by **X**_*i*_, as follows:

(2) $$\begin{array}{c} X_{i}=\frac{Y_{i}-\text{Ave}\left(Y_{i}\right)}{S_{i}}, \end{array}$$

where Ave(**Y**_*i*_) is the average value of the components of **Y**_*i*_ and *S*_*i*_ is the standard error of the components of **Y**_*i*_. The standardization is needed to unify the variances of the time series, and the unified variances can be led to comparable spectrum densities across profiles. Note that the standardization does not change the behavior of the time series. Significant linear trends in experimental data are observed broadly. They are not biologically meaningful but may affect the periodicity estimate. So a linear regression model is then fitted to **X**_*i*_ to remove the linear trend from the time series, and the detrended time series is denoted by ${\dot{X}}_{i}$. The Savitzky-Golay (S-G) smoothing filter [20] with order 4 is then applied to ${\dot{X}}_{i}$ in order to reduce the noise level without much biasing the data, and the resulting new time series is represented by ${\ddot{X}}_{i}$. In an autoregressive model with order of *d*, denoted by AR(*d*), the current state of a time series is assumed to depend on the previous *d* states only. Since the longest period of interest in this study is 24 hours and the method is designed for 4-hour temporal resolution data, it is reasonable to consider an AR(6) model, in which the gene expression levels within the previous 24 hours are considered. Both ${\dot{X}}_{i}$ and ${\ddot{X}}_{i}$ are modeled via an AR(6) process of order 6 and model parameters are estimated by each of the following three methods: Yule-Walker method [21, 22], Burg method [23], and maximum likelihood estimation (MLE) [24]. Thus six AR models, (*M*_*i*1_,…, *M*_*i*6_), for each manipulated gene expression profile are obtained. For each AR model, spectral analysis is then applied to obtain one set of frequencies along with their spectral densities. Unlike ARSER, all frequencies and their corresponding spectral densities are considered to estimate the period and classify the genes according to their periods into three categories: arrhythmic, ultradian, and circadian.

Next, let the six sets of frequencies obtained by fitting the AR models of the *i*th gene expression profile be *Ω*_*ij*_  (*j* = 1,…, 6). For the *j*th set of frequencies, a harmonic model *H*_*ij*_ is considered as follows:

(3) X˙it=μij+∑k=1Kijpijkcos⁡2πωijkt+qijksin⁡2πωijkt+ϵijt,

where ${\dot{X}}_{it}$ is the detrended profile of the *i*th gene at time *t*, *μ*_*ij*_ is the constant term of the *j*th harmonic model for ${\dot{X}}_{it}$, (*ω*_*ij*1_,…, *ω*_*ijK*_*ij*__) are the elements of the frequency set *Ω*_*ij*_ provided that there are *K*_*ij*_ elements in that set, (*p*_*ij*1_,…, *p*_*ijK*_*ij*__, *q*_*ij*1_,…, *q*_*ijK*_*ij*__) are unknown linear parameters of the trigonometric terms, and *ϵ*_*ijt*_ is the error term for the *i*th gene *j*th harmonic model at time *t*. The posterior probabilities of the six harmonic models are estimated and the model with the largest posterior probability is selected as the optimal model. A period is defined as the dominant period if it corresponds to the highest peak of the frequency spectrum of the optimal model.

**Figure 1:**
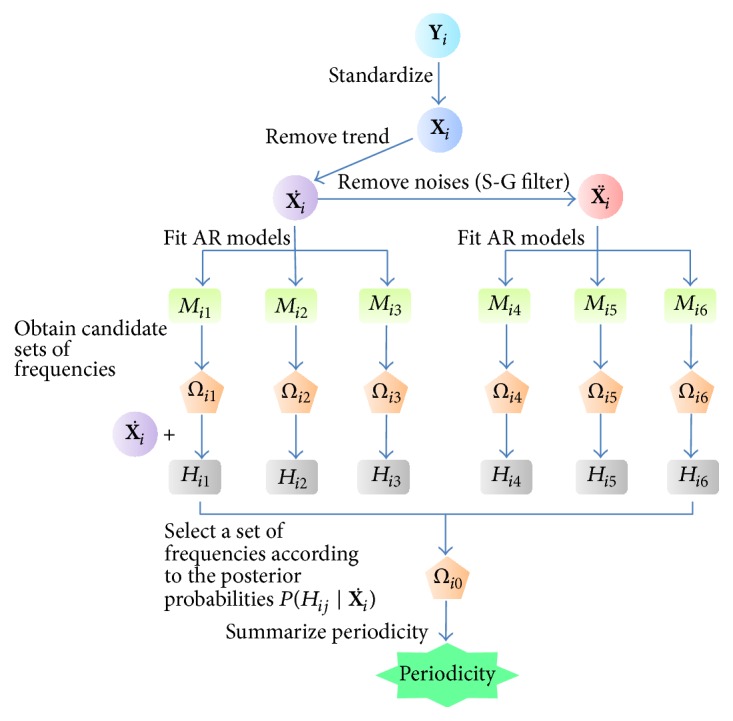
Flowchart of ABSR algorithm. **Y**_*i*_ is the observed profile of the *i*th gene expression and **X**_*i*_ is the standardized time profile from **Y**_*i*_. ${\dot{X}}_{i}$ is the detrended profile derived from **X**_*i*_ and ${\ddot{X}}_{i}$ is the S-G filtered profile from ${\dot{X}}_{i}$. The AR models *M*_*i*1_, *M*_*i*2_, and *M*_*i*3_ are fitted for the detrended data ${\dot{X}}_{i}$ by three methods of model parameter estimation: Yule-Walker method, Burg method, and MLE, respectively. The AR models *M*_*i*4_, *M*_*i*5_, and M_*i*6_ are fitted for the noise-reduced data ${\ddot{X}}_{i}$ by the three above-mentioned model parameter estimation methods, respectively. The frequency sets (*Ω*_*i*1_,…, *Ω*_*i*6_) are obtained from the frequency spectra of the AR models (*M*_*i*1_,…, *M*_*i*6_), respectively. The harmonic models *H*_*i*1_,…, *H*_*i*6_ are constructed with ${\dot{X}}_{i}$ and the candidate frequency sets (*Ω*_*i*1_,…, *Ω*_*i*6_). *Ω*_*i*0_ is the selected frequency set.

Lastly, each gene is classified according to the criteria described in Section 2.3.  Figure 1 shows a flowchart describing the ABSR algorithm.

### 2.2. Model Selection

Model selection in ABSR proceeds by estimating the posterior probability of each harmonic model and then selecting the model with the largest posterior probability as the optimal model. To calculate a posterior probability, model (3) is presented in the matrix form:

(4) $$\begin{array}{c} {\dot{X}}_{i}=G_{ij}\beta_{ij}+\epsilon_{ij}, \end{array}$$

where *G*_*ij*_ = (1, *G*_*ij*1_^*c*^, *G*_*ij*1_^*s*^,…, *G*_*ijK*_*ij*__^*c*^, *G*_*ijK*_*ij*__^*s*^) and

(5) 1=11⋮1,Gijkc=cos⁡2πωijkcos⁡4πωijk⋮cos⁡2Tπωijk,Gijks=sin⁡2πωijksin⁡4πωijk⋮sin⁡2Tπωijkk=1,2,…,Kij,βij=μijpij1qij1⋮pijKijqijKij,ϵij=ϵij1ϵij2⋮ϵijT.

Normal and inverse gamma distributions are assumed as prior distributions for the parameters and hyperparameters as follows:

(6) μij~Nμi,σμ2ij,pijk~N0,σp2ijk,qijk~N0,σq2ijk,ϵijt~N0,σϵ2ij,σμ2ij~IG2,σi2,σp2ijk~IG2,σi2,σq2ijk~IG2,σi2,σϵ2ij~IG2,σi2,

where *μ*_*i*_ and *σ*_*i*_^2^ are the sample mean and sample variance of the components of the *i*th detrended profile (*i* = 1,…, *N*; *j* = 1,…, 6; *k* = 1,…, *K*_*ij*_; *t* = 1,…, *T*). All parameters and hyperparameters are assumed to be independent. It follows that the conditional distribution of ${\dot{X}}_{i}$ given **β**_*ij*_ follows the normal distribution *N*(*G*_*ij*_ **β**_*ij*_, Σ_*ij*1_), where Σ_*ij*1_ = Diag ((*σ*_*ϵ*_^2^)_*ij*_,…, (*σ*_*ϵ*_^2^)_*ij*_). Here Diag (**a**) indicates a diagonal matrix with the diagonal vector **a**. The union of all the parameters and hyperparameters is denoted by ***θ***_*ij*_.

In the absence of any reason to prefer one model over the others, it is reasonable to assume equal prior probability for each model; namely, *P*(*H*_*ij*_) = 1/6. Hence the posterior probability *P*(*H*_*ij*_∣**X**_*i*_) is proportional to (with same rate for all *j*'s) the likelihood function of the data ${\dot{X}}_{i}$ given the harmonic model *H*_*ij*_, namely, $Pr({\dot{X}}_{i}|H_{ij})$. Instead of directly calculating the posterior probabilities, the likelihood function $Pr({\dot{X}}_{i}|H_{ij})$ is calculated. The likelihood function $Pr({\dot{X}}_{i}|H_{ij})$ can be written as the integral of the product of the likelihood function $Pr({\dot{X}}_{i}|\theta_{ij},H_{ij})$ and the probability density function of the prior distribution Pr(***θ***_*ij*_∣*H*_*ij*_) with respect to the parameter vector ***θ***_*ij*_,

(7) $$\begin{array}{c} Pr\left(\;{\dot{X}}_{i}\;|\;H_{ij}\;\right)=\int Pr\left(\;\theta_{ij}\;|\;H_{ij}\;\right)Pr\left(\;{\dot{X}}_{i}\;|\;\theta_{ij},H_{ij}\;\right)d\theta_{ij}. \end{array}$$

This integral can be simplified as

(8) ∫⋯∫NX˙i;Gijβij0,GijΣij2Gij′+Σij1·Prσμ2ij,σp2ij1,σq2ij1,…,σq2ijKij ∣ Hijdσμ2ij⋯dσq2ijKij,

where **β**_*ij*0_ = (*μ*_*i*_, 0,…,0)′, Σ_*ij*2_ = Diag ((*σ*_*μ*_^2^)_*ij*_, (*σ*_*p*_^2^)_*ij*1_, (*σ*_*q*_^2^)_*ij*1_,…, (*σ*_*q*_^2^)_*ijK*_*ij*__, (*σ*_*q*_^2^)_*ijK*_*ij*__), and *N*(**a**; **b**, *C*) is the probability density function of the multivariate normal distribution with mean **b** and covariance matrix *C* with respect to **a**.

This integral cannot be simplified further but can be estimated by Monte Carlo method. The steps of model selection procedure are as follows:

(1) Simulate each variance parameter according to its prior distribution.
(2) Calculate the value of the likelihood function $N({\dot{X}}_{i};G_{ij}\beta_{ij0},G_{ij}\Sigma_{ij2}G_{ij}^{\prime }+\Sigma_{ij1})$.
(3) Repeat steps (1) and (2) 10,000 times and then take the average of the 10,000 likelihood function values. This average value is an estimate of the integral.
(4) Repeat steps (1) through (3) for all six models and choose the model with the largest estimate of the integral.

### 2.3. Criteria of Rhythmic Categories

Given the optimal model, as determined by maximizing the posterior probability, the following values can be calculated: the highest peak of the spectral densities, the *p* value of the *F*-test for the corresponding period, and the estimate of the dominant period. Yang and Su [17] apply Storey and Tibshirani's approach [25] to calculate the *q*-values to determine the significance of a period. However, when the *p* values are not distributed in the full range of [0,1], Storey and Tibshirani's *q*-value may not be appropriate. For this reason, Benjamini-Hochberg (BH) [26] *q*-value is applied in this proposed method. According to our simulation study, it is found that the maximum value of the spectral densities of a noisy signal is on average less than that of an oscillating signal with the same variance. Therefore, a threshold for the spectral density is considered and a gene is assigned into one of the rhythmic categories (ultradian, circadian, and arrhythmic) according to the following sorted criteria:

(i) If the maximum value of the spectral densities is less than a preselected spectrum threshold (e.g., 10 or 5) or the dominant period is not significant (*q*-value ≥ 0.05), the gene is classified as arrhythmic.
(ii) Otherwise, the gene is classified by the estimate of the dominant period. User-defined intervals for ultradian and circadian categories are used to classify the profiles. In particular, the rhythmic categories are defined as follows: if the estimated period is greater than or equal to 6 hours and strictly less than 10 hours, denoted by the time interval of [6,10), the gene is classified as ultradian8. Similarly, the gene is classified as ultradian12 for the period interval of [10,14) and as circadian for [20,28). All the other genes are classified as arrhythmic.

The value of the spectrum threshold needs to be selected. By calculating the number of rhythmic profiles for each value of the spectrum threshold in consideration (e.g., from 10 to 0 with step of 0.5), the correspondence of the number of rhythmic profiles and the value of the spectrum threshold can be studied, and the value of the spectrum threshold can be selected according to prior knowledge and research purpose. For example, if the research goal is to discover as many rhythmic genes as possible, then the value of threshold with the maximum number of rhythmic profiles can be selected. In the case of searching for a less conservative result, the spectrum of threshold can be selected to be the largest value that the number of rhythmic profiles does not change significantly as the threshold value reduces. If one can assume the data with less noise or is interested in conservative detection of rhythmic profiles, a large value of the threshold could be applied. For example, a threshold of 10 is used in the simulation studies.

## 3. Results and Discussion

### 3.1. Simulation Study

#### 3.1.1. Periodicity Estimate for Fixed Period Settings

To assess the performance of the ABSR algorithm, sequences of sinusoidal data to represent profiles with a length of 48 hours that consists of 4-, 2-, or 1-hour resolution are generated. Four periodic behaviors are considered: periods of 8 and 12 hours (ultradian rhythms), period of 24 hours (circadian rhythm), or aperiodic (arrhythmic profiles). It is noticed that gene expression profiles with a linear trend are common in the experimental data, so both patterns of cosine function with and without a linear trend (Table 1) are considered. For each combination of resolution and period, 1,000 sequences are simulated, among which 500 sequences are cosine waves, and the other 500 sequences are cosine waves with a linear trend. The amplitude is set to be 5.0 and standard normal error is integrated to the data. These simulated data can be downloaded from the website http://homepages.uc.edu/~songso/. JTK_CYCLE and ARSER are also applied to the simulated data to compare their performances with ABSR.

In order to describe the performance of the three algorithms, the following five terms are defined. A* discovery* implies that a gene is classified to be ultradian or circadian. A discovery is a* true discovery* if a gene is classified as its true category. The percentage of genes within a category (either ultradian or circadian) that are classified correctly is called the* true discovery rate* (TDR). For example, in our simulation study, the TDR of the circadian category is the percentage of all 1,000 circadian profiles classified as circadian. A discovery is a* false discovery* if a gene is discovered but classified as a category other than its true category. The* false discovery rate* (FDR) is the percentage of all discoveries of a category that are false discoveries. Notice that since in this study four categories, instead of typically binary decisions, are considered, the definition of FDR here is different from the classical definition. Higher TDR implies higher ability to detect oscillations, and lower FDR implies higher reliability in discovering rhythmic gene expression.

The two ultradian datasets (with true period = 8 and 12) are denoted by ultradian8 and ultradian12, respectively, and the combined dataset of the 4,000 profiles from the four categories (arrhythmic, ultradian8, ultradian12, and circadian) is used to calculate TDRs and FDRs. To make this comparison reasonable, the window of period of 6 to 28 hours (8 to 28 hours for 4-hour resolution) is considered for JTK_CYCLE, and the period windows of 6 to 14 hours and 20 to 28 hours are considered for ARSER. Since ARSER and JTK_CYCLE estimate the period of gene expression but do not classify genes into rhythmic categories, comparing of the classification is done based on the period estimates and their significance. A profile is considered as ultradian8 if its period estimate is significant (*q*-value less than 0.05) and within the interval of [6,10), ultradian12 for [10,14), and circadian for [20,28). ARSER may provide more than one significant period estimate and these estimates may fall in different windows of interest. In such case the classification of the profile is not definite and is denoted by “undefined.”

Table 2 shows the comparisons of classification among ABSR, ARSER, and JTK_CYCLE for the data with 4-, 2-, and 1-hour temporal resolutions. Columns 3 to 6 of the table contain the numbers of profiles that are in the corresponding intersection of categories. Across all resolutions, ABSR obtains high TDR of more than 90% and low FDR of less than 8%, while JTK_CYCLE shows low TDR with 4-hour resolution and ARSER classifies a large portion of the profiles as undefined.

Among the 539 (bold in Table 2) discovered circadian profiles with 4-hour resolution by JTK_CYCLE, 426 profiles are found without linear trend, and the other 113 are with a linear trend. It is also observed that as the proportion of profiles with a linear trend increases, TDR of circadian profiles tends to decrease by JTK_CYCLE. However, ABSR provides the results unchanged.

**Figure 2:**
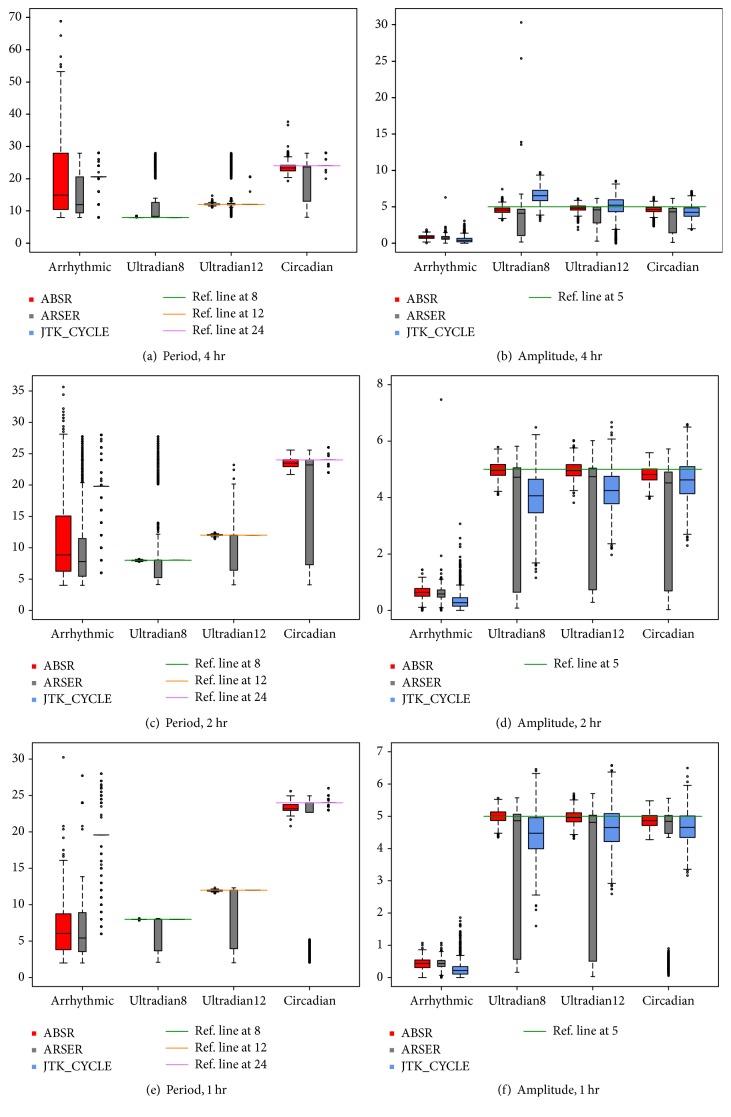
Boxplots of period and amplitude estimate for data with different temporal resolutions. Three outliers (one in each rhythmic category by ABSR) are excluded from (a). One arrhythmic profile by ABSR is excluded from (c). Forty-five arrhythmic profiles by ABSR are excluded from (e). Those outliers represent infinitely large period estimates, which imply arrhythmic property.

In addition to the TDRs and FDRs of periods, Figure 2 shows boxplots of period and amplitude estimates by each method applied to the simulated data with 4-, 2-, and 1-hour resolutions. The reference lines show true values of periods and amplitudes, and the black bold bar inside each box indicates the median estimate for the corresponding rhythmic profiles.

Although the period estimates by JTK_CYCLE with 4-hour resolution are shown to be less biased, the majority of the estimates are not statistically significant. On the other hand, ABSR results in significant period estimate for more than 90% of the rhythmic profiles with the bias of at most 0.55. Notice the circle above the JTK_CYCLE box of the ultradian12 profiles represents 63 ultradian12 profiles, while the bench of circles above and under the ABSR box represents 36 profiles. The standard error by ABSR is slightly greater than by JTK_CYCLE (2.64 versus 2.08). Since ARSER provides period estimate in diverse windows for a large portion of data, the standard errors by ARSER are much larger than by ABSR and JTK_CYCLE for various rhythmic categories with various resolutions.

Considering the amplitude estimate, ABSR performs better with less bias and smaller standard error than ARSER and JTK_CYCLE for all categories and temporal resolutions.

**Figure 3:**
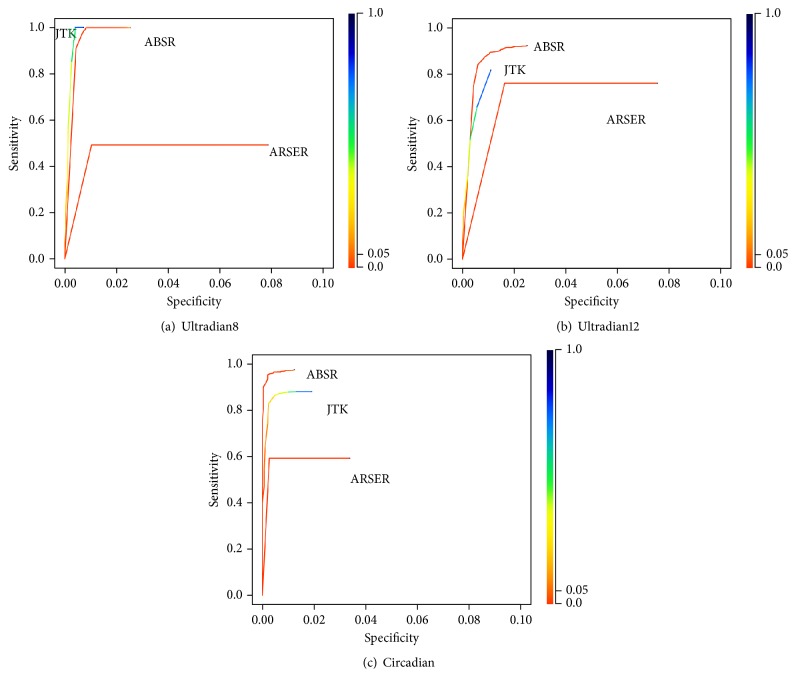
ROC plots for data with 4-hour resolution.

Figure 3 shows the receiver operating characteristic (ROC) curves for the three rhythmic categories. In these plots, the test is done for binary decision of categories: rhythmic (ultradian8, ultradian12, and circadian) and arrhythmic. Since the period estimate is taken into consideration when testing the rhythmicity, the specificity is far below 1 in this study. The color represents the *q*-value threshold used to calculate the sensitivity and specificity. The plots for ultradian12 and circadian categories show clearly that ABSR performs better than the other two algorithms. In the plot for ultradian8 category, JTK_CYCLE shows higher sensitivity and lower specificity than ABSR but with large *q*-value threshold. With typically used *q*-value threshold (0.05), JTK_CYCLE is not sensitive (sensitivity = 0.036).

#### 3.1.2. Periodicity Estimate for Random Periodicity Settings

**Figure 4:**
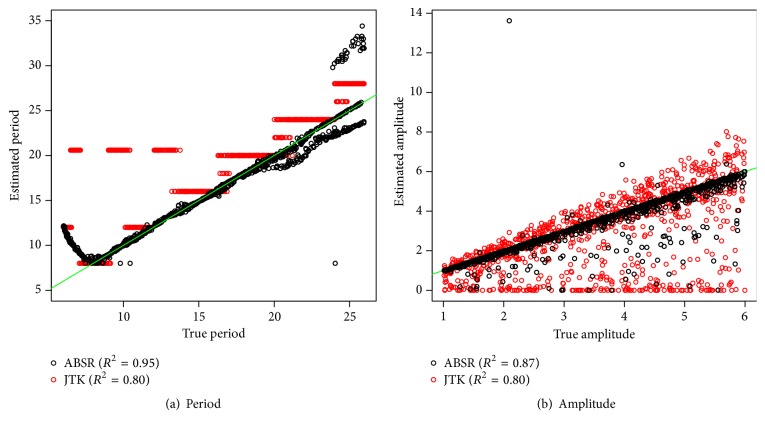
Period and amplitude estimate for randomized period. Fifty-eight outliers with huge period estimate by ABSR are excluded from (a). The green reference line is with the slope of 1.

In the above-mentioned simulation study, three fixed values of period, amplitude, phase, and signal/noise ratio are considered. To assess the performance of ABSR on more flexible parameter settings, two more simulation studies are performed. Since both ABSR and JTK_CYCLE provide one single periodicity estimate for one profile, comparison between ABSR and JTK_CYCLE only is performed. In the first simulation, 1000 extra profiles are generated with uniformly distributed periods, amplitudes, and phases. Periods are within 6 to 26 hours, amplitudes are within 1 to 6, and phases are within 0 to the corresponding period. Again the profiles are simulated for 48-hour course with 4-hour resolution. Standard normal errors are added to the sinusoidal waves. ABSR considers all positive values for the period estimate, but very large estimates are not of interest. Hence 58 profiles with very large period estimate (>35 hours) are removed, and comparison of period and amplitude estimates with JTK_CYCLE is done. By providing continuous period estimates, ABSR shows stronger linear correlation than JTK_CYCLE for both period and amplitude estimates (Figure 4). In the range of 8 to 20 hours for the true period, ABSR clearly provides less biased period estimate than JTK_CYCLE and, in the range of 20 to 24, both ABSR and JTK_CYCLE may provide a period estimate with a bias.

**Figure 5:**
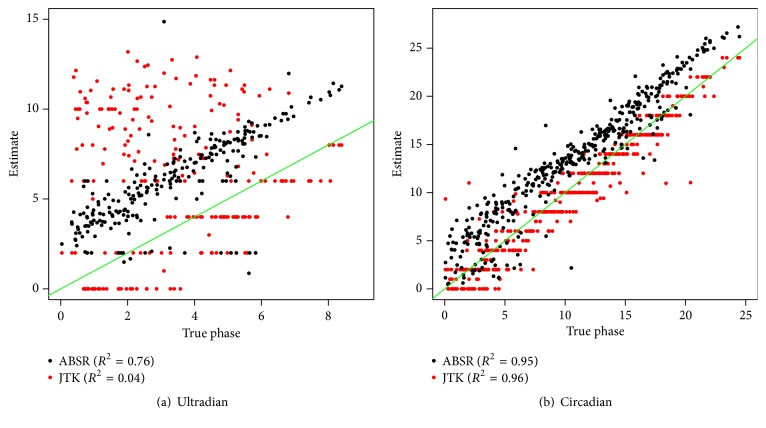
Phase estimate for randomized rhythmicity.

Besides period and amplitude estimates, the phase information is also an important aspect of rhythmicity. To clearly show the performance of phase estimate, in the second study, 500 ultradian profiles and 500 circadian profiles are simulated. The profiles are generated with sinusoidal pattern with the parameters uniformly distributed: period from 8 to 12 for ultradian profiles and from 22 to 26 for circadian profiles, amplitude from 1 to 6, linear slope from −0.1 to 0.1, and phase from 0 to the length of the cycle. Standard normal error is added to each profile. It is noticed that when the true phase is close to zero or the true period, both ABSR and JTK_CYCLE sometimes result in a noticeable bias in phase estimate. This may be caused by the low temporal resolution. By removing those profiles, it is found that the correlation coefficients are similar by ABSR and JTK_CYCLE for circadian profiles, but much higher by ABSR than by JTK_CYCLE for ultradian profiles (Figure 5).

The settings in the first study provide the broad testing of wide range of period and different ratios of the amplitude over noise, and the settings in the second study provide the broad testing of wide range of phase. It is found that ABSR performs well in both studies, so it can be used in diverse situations.

#### 3.1.3. Detection of Circadian Rhythms for Nonsinusoidal Patterns

**Figure 6:**
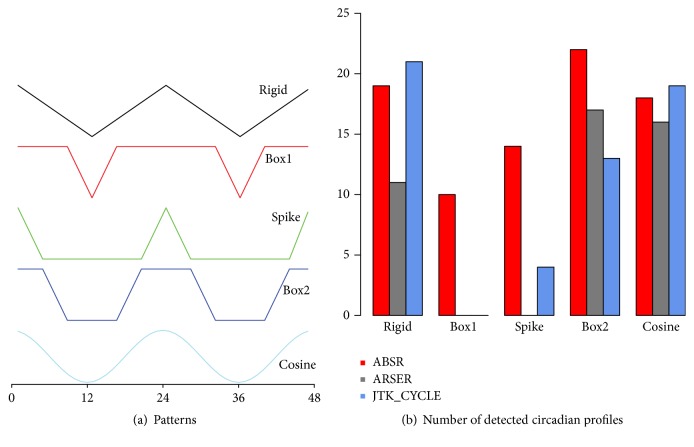
Detecting circadian rhythms for nonsinusoidal patterns.

Though cosine wave is typically assumed, some experimental data exhibits nonsinusoidal pattern. So a good method should be able to detect the rhythms for nonsinusoidal patterns as well. The performance of ABSR to detect nonsinusoidal circadian rhythms when both ultradian and circadian rhythms are of interest is then assessed. Five different circadian (period = 24) patterns [17, 27] (rigid, spike, two box-like patterns and cosine wave) are considered (Figure 6(a)). Twenty-four profiles are generated from each pattern, adding standard normal error, with hourly lag from 0 to 23. Again the same ultradian and circadian windows are applied as in previous simulation studies for ARSER and JTK_CYCLE.  Figure 6(b) shows the number of detected circadian rhythms by each algorithm. It is found that, among the five patterns, all three algorithms perform well for rigid, box2, and cosine patterns. For the patterns of box1 and spike, ABSR detects 10 and 14 out of 24 circadian rhythms, respectively, whereas ARSER and JTK_CYCLE can hardly detect any circadian rhythm. This implies that ABSR is more robust and insensitive to rhythmic patterns, in general.

From the above-mentioned simulation studies, it is found that ABSR performs best among the three algorithms with low resolution (4-hour) by being highly sensitive in detecting rhythmic profiles with low FDR and produces period, amplitude, and phase estimates which are close to the true values independent of the temporal resolution. ABSR is capable of discovering harmonic ultradian and circadian profiles simultaneously, and the performance is not affected by the proportion of profiles with a linear trend. As the temporal resolution increases, ABSR and JTK_CYCLE perform better with respect to FDR and TDR, but JTK_CYCLE is more beneficial in high temporal resolution.

### 3.2. Application to Experimental Data

Hughes et al. [28] performed experiments on mouse livers (GSE11923) to observe transcriptional oscillations with high accuracy of 1-hour temporal resolution within 48-hour time-course and found the existence of harmonics of circadian gene expression in mice. They argued that the increase of sampling resolution of rhythmic gene profiles allows detecting cycling genes better as compared to experimental data with 4-hour temporal resolution, which is typical in gene expression profiling. To explore the performance of the ABSR on the typically designed experimental data, the data is coarsened with 4-hour temporal resolution by selecting a subset of the original data for every 4 hours, and ABSR, ARSER, and JTK_CYCLE algorithms are applied to the coarsened data for comparison.

**Figure 7:**
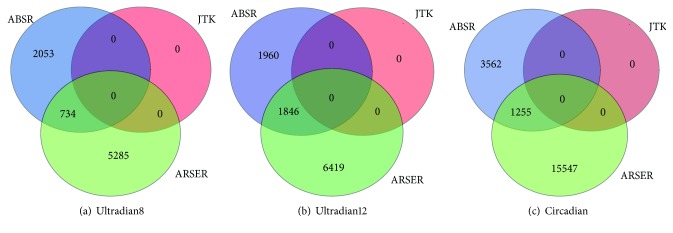
Classification of rhythmic categories for the mouse liver data with 4-hour resolution.

Spectrum thresholds from 0 to 10 with increment of 0.5 are considered, and since the goal is to discover as many rhythmic genes as possible, the threshold of 2.5 is selected.  Figure 7 shows the classification of circadian and ultradian categories. JTK_CYCLE is not able to detect either circadian or ultradian profiles; however, ABSR discovers 2,787 ultradian8, 3,806 ultradian12, and 4,817 circadian profiles and ARSER discovers 6,019 ultradian8, 8,265 ultradian12, and 16,802 circadian profiles.

**Figure 8:**
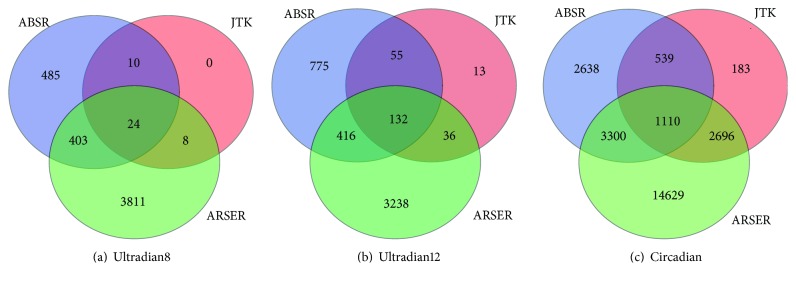
Classification of rhythmic categories for the mouse liver data with 1-hour resolution.

**Figure 9:**
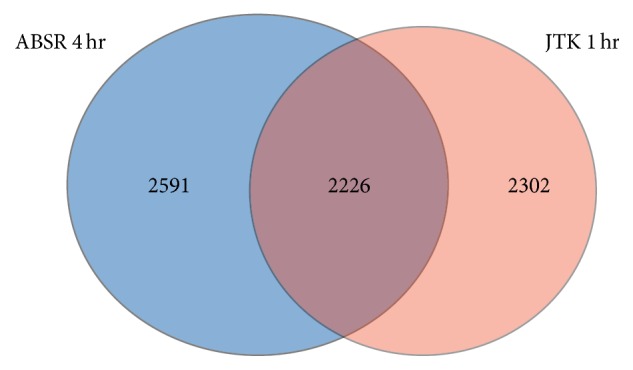
Comparison for discovered circadian profiles between ABSR with 4-hour resolution and JTK_CYCLE with 1-hour resolution.

In addition, the three algorithms are applied to the original data, and the spectrum threshold of 1 is selected.  Figure 8 shows the classification results. With 1-hour resolution, JTK_CYCLE captures 4,528 circadian profiles. It is found that, among the 4,817 circadian profiles classified by ABSR from the data with 4-hour resolution, 2,226 profiles are classified as circadian by JTK_CYCLE from the data with 1-hour resolution (Figure 9). Therefore, 49.2% of the circadian profiles detected by JTK_CYCLE with 1-hour resolution are also detected by ABSR with 4-hour resolution.

**Figure 10:**
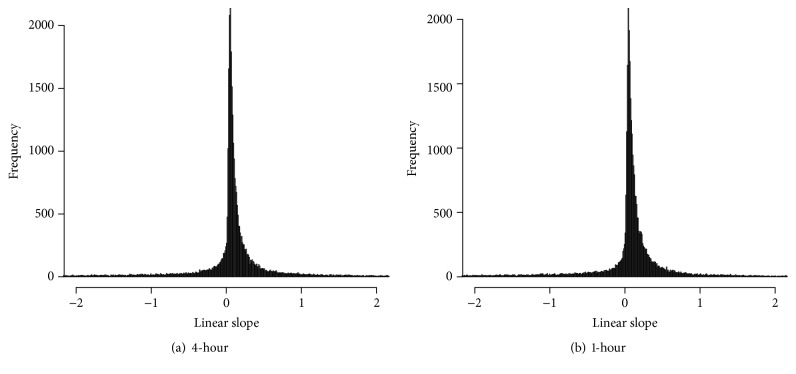
Histogram of linear slope for mouse liver data with different temporal resolution.

To further understand the result, the linear trend in each profile for both temporal resolutions is examined.  Figure 10 shows the distribution of the linear slopes of the profiles for the mouse liver data with both 1- and 4-hour resolutions. Among the 45101 profiles, 67% and 68% of the profiles are with a linear slope more than 0.1 far away from 0 for the data with 4- and 1-hour resolutions, respectively. As found in the simulation study, ABSR is not affected by the proportion of profiles with a linear trend, but when the time series is short, JTK_CYCLE discovers fewer rhythmic profiles as the proportion of profiles with a linear trend increases. Hence, for experimental short time-course data, ABSR can be a more appropriate algorithm to detect rhythms.

## 4. Conclusions

In this paper, we present a new algorithm, ABSR, to determine the rhythmicity of a gene expression profile with short time series. For noisy short time series (e.g., profiles within 48 hours with 4-hour resolution), ABSR performs well in estimating period and amplitude and substantially reducing the FDR of ARSER and increasing the TDR of ARSER and JTK_CYCLE. To apply the JTK_CYCLE algorithm, a user-defined window of period is required, and it is observed that different user-defined windows might obtain inconsistent estimates. However, there is no such constraint in ABSR, and the estimates are consistent even with sparse observing temporal resolution relative to the true period. Moreover, the single period estimate without a preset window enables ABSR to discover any harmonic and circadian rhythms simultaneously. Since ABSR manipulates the data to treat the linear trend and unwanted noise, ABSR can be applied to data with less consideration of the quality. Inheriting from ARSER, ABSR is also a joint strategy to analyze data through both frequency and time domains. Though experiments with duration of more days and high resolution may help us study the rhythms better, the cost and feasibility are not always realistic. Due to the cost of experiments, most of the time-course experiments designed to study rhythms are performed for 48 hours with 4-hour resolution. In this particular case, ABSR is a better choice, and, with the tunable thresholds, the trade-off can be small.

Since ABSR assumes continuous values for the period estimate, it can estimate any rhythms, not limited to ultradian or circadian rhythms. Estimating the period is the first step, and classification is the second step. If one is only interested in the first step, the classification step can be skipped.

In this study, the longest period in consideration is 24 hours, and the temporal resolution is focused on the typical 4-hour resolution, so the AR(6) model is used to obtain candidate periods. For other experimental design settings, the ABSR model can be extended to another order, where the order is in the form of longest period of interest/temporal resolution.

The value of threshold for the spectral density may affect the classification results, so the choice of threshold is crucial. As a consequence of choosing a large threshold, the results could be conservative. In other words, some rhythmic profiles might not be detected, while the detected rhythmic profiles could be accepted with more confidence.

Since ABSR is a Bayesian algorithm, inevitably, the computing time is a concern. The likelihood functions are estimated independently across different profiles, so the data can be partitioned and the algorithm can run in parallel to increase the computational efficiency. Our computer is a workstation with technical specification as Intel Xeon E5-2687W (2 processors), 3.10 GHz, 256 GB RAM, Windows 7 Ultimate, and R version 3.1.2. The computation efficiency is tested with 4-, 2-, and 1-hour temporal resolutions within 48-hour time-course data. Running the algorithm with 30 threads in parallel, it is observed that, for one single thread, 3 to 4 profiles are analyzed per minute for the 4-hour resolution data, 2 to 3 profiles are analyzed per minute for the 2-hour resolution data, and about 2 profiles are analyzed per minute for the 1-hour resolution data.

Although ABSR performs best among the three algorithms for short noisy time series, it is not the best choice for all situations. For example, ABSR is useful for users who would like to maximize the discovery of rhythmic genes with 4-hour temporal resolution data. As the length of the time series increases, the number of parameters to be sampled in estimating the posterior probability also increases, so the convergence of the estimate could be a concern. In case of long time series, JTK_CYCLE would be a better choice to identify the classification of time-course gene expression profiles rather than ABSR. Therefore, users will need to choose an optimal algorithm based on their experimental conditions.

## Figures and Tables

**Table 1:** Formula used to simulate data.

Pattern	Function
Noise	*y*_*t*_ = *ϵ*
Cosine	*y*_*t*_ = *A* · cos(2*πt*/*p* + *ϕ*) + *ϵ*
Noise with linear trend	*y*_*t*_ = *C* · *t* + *ϵ*
Cosine with linear trend	*y*_*t*_ = *C* · *t* + *A* · cos(2*πt*/*p* + *ϕ*) + *ϵ*

*A* = 5; *p* = 8, 12, 24; *ϕ* = 0.5; *ϵ* ~ *N*(0,1); *C* = 0.1.

**Table 2:** Classification comparisons for fixed period data.

Resol.	Result category	True category				TDR	FDR
		Arrhy.	Ultra.8	Ultra.12	Circa.	(%)	(%)
	*ABSR *						
	Arrhy.	818	1	75	29	—	—
	Ultra.8	72	999	0	0	99.9	6.7
	Ultra.12	76	0	925	0	92.5	7.6
	Circa.	34	0	0	971	97.1	3.4
	*ARSER*						
	Arrhy.	0	0	0	0	—	—
	Ultra.8	236	493	0	0	49.3	32.4
4 hr	Ultra.12	226	0	760	0	76.0	22.9
	Circa.	101	0	0	592	59.2	14.6
	Undef.	437	507	240	408	—	—
	*JTK_CYCLE*						
	Arrhy.	998	964	943	461	—	—
	Ultra.8	0	36	0	0	3.6	0.0
	Ultra.12	0	0	57	0	5.7	0.0
	Circa.	2	0	0	**539**	53.9	0.4

	*ABSR*						
	Arrhy.	993	0	1	0	—	—
	Ultra.8	6	1000	0	0	100.0	0.6
	Ultra.12	0	0	999	0	99.9	0.0
	Circa.	1	0	0	1000	100.0	0.1
	*ARSER*						
	Arrhy.	67	0	0	0	—	—
	Ultra.8	241	487	0	0	48.7	33.1
2 hr	Ultra.12	71	0	413	0	41.3	14.7
	Circa.	80	0	0	383	38.3	17.3
	Undef.	541	513	587	617	—	—
	*JTK_CYCLE*						
	Arrhy.	993	0	0	0	—	—
	Ultra.8	2	1000	0	0	100.0	0.2
	Ultra.12	3	0	1000	0	100.0	0.3
	Circa.	2	0	0	1000	100.0	0.2

	*ABSR*						
	Arrhy.	1000	0	0	0	—	—
	Ultra.8	0	1000	0	0	100.0	0.0
	Ultra.12	0	0	1000	0	100.0	0.0
	Circa.	0	0	0	1000	100.0	0.0
	*ARSER*						
	Arrhy.	201	0	0	0	—	—
	Ultra.8	139	496	0	0	49.6	21.9
1 hr	Ultra.12	115	0	572	0	57.2	16.7
	Circa.	80	0	0	776	77.6	9.3
	Undef.	465	504	428	224	—	—
	*JTK_CYCLE*						
	Arrhy.	996	0	0	0	—	—
	Ultra.8	1	1000	0	0	100.0	0.1
	Ultra.12	1	0	1000	0	100.0	0.1
	Circa.	2	0	0	1000	100.0	0.2
